# Twin Studies in the Danish Cancer Registry, 1942-55

**DOI:** 10.1038/bjc.1957.40

**Published:** 1957-09

**Authors:** A. Nielsen, J. Clemmesen


					
BRITISH JOURNAL OF CANCER

VOL. XI           SEPTEMBER, 1957           NO. 3

TWIN STUDIES IN THE DANISH CANCER REGISTRY, 1942-55

A. NIELSEN AND J. CLEMMESEN

From the Danish Cancer Registry under the National Anti-Cancer League,

Strandboulevarden 49, Copenhagen, Denmark

Received for publication June 20, 1957

THE Danish Cancer Registry, at its opening in 1942 initiated a joint effort with
the University Institute for Human Genetics in Copenhagen, which subsequently
resulted in monographs on heredity of breast cancer by Jacobsen (1946), and of
uterine cancer by Br0beck (1949), and later in separate studies from the two
institutes by Busk (1948, 1952) and Videbaek (1947), on leukaemia. Later the
Genetical Institute took interest in gastric carcinoma and allied problems (Videbaek
and Mosbech, 1954; Mosbech, 1953) while the Cancer Registry continued its
studies on twins with cancer, begun by Busk, Clemmesen and Nielsen (1948),
and brought up to date with the present publication.

MATERIAL

In the period from January 1942 to June 1955 inclusive, 140,000 new cases of
cancer were filed at the Cancer Registry of which about 110,000 were notified
from hospitals, and the remaining 30,000 known from death certificates only.
Notification forms contain the question whether the patient is a twin-and this
was answered for in 73 per cent of cases. When the answer was in the affirmative
a separate notification of the twin sib was filled in and paid by a symbolic fee of
10 kroner, equivalent to 10 shillings. This special notification gives the data of
the twin sib together with information on previous disease of more serious character
including diagnosis and the date and place of treatment. In order to decide
whether the twins are monozygotic or dizygotic the form contains questions about
similarity and about mistakes on identity made by parents and others. Other
questions refer to colour of hair, colour of eyes, stature, height and weight. Almost
full congruity is demanded before a pair is accepted as monozygotic. The authors
have not been in a position to contact patients or their twins personally, but have
trusted information from hospital physicians.

As shown in Table II, 849 pairs of twins were notified during the period studied
(a), and the twins were grouped according to identity of sex and similarity of
appearance. Exclusion of 178 pairs (b) was necessary because of insufficient
information, mostly due to extreme weakness or death of one partner, and in
some cases because it proved impossible to decide whether or not the pair were
identical twins.

The first partner of a pair to be notified as suffering from cancer during the
period studied was signified as I while the other partner would be classified as II.

22

A. NIELSEN AND) J. CLEMMESEN

0

4-4

0

0 0

z z

O d-1

W  .o8

?

344

0

C)-

0
za 0

04

10 a) l  rc: 41 eO  ~OC O - -o r

I~~0~~  C~~~ ~    0
-  ?t 1k -4 o   0o) ol  o o
cXi  e XO CI  C LE oJcq~ s  O>> Xo

-  --

* . .   .   .   ..

- se o O cs  0 o-A  5ao o - eO

o0 o                      o? C V X  0 e  C
_1 0 C)  c>  OD  aq CC  c lo C > e  C:

-o  i  sXsO~ ft  r      O b  0 s+ :

aq              eq oq ct  m  m
00 O)  _   aq  t- 00  a q * C>  00 o

to    I I o o co  co  - o  o  _,

c          o c o c- =  _ w oo OD  41 ?  0

-6~~~ ~~ Ci U4C 6COs*e Q

0

O'~

e~ ~~c Ca O o

?l(  :0 0 a

.    .   .   .   .   . .   .   .   .. *

0~0

1*

10

.~~~~~~~~~0~~.

_

r-   00  ( 3-00   0'

*   *   *   *   I L *  _e

--------- --  -  cq o;

10

o

CO

0

I

o
cq

328

00
I.
i4.i

pq

*D

TWIN STUDIES IN DANISH CANCER REGISTRY

In 38 cases twin II had left Denmark (c), and the pair had to be discarded due
to insufficiency of information. For the same reason we had to exclude 191 pairs
(d) of which twin II had died aged under five years and before the registry opened
in 1942. The few casualties of this category occurring after that date could be
traced by means of death certificates.

Those pairs which had not been notified as twins when the first partner
developed cancer would naturally have to be discarded, even if they were notified
when a tumour developed in the second partner, because only pairs with cancer in
both partners-the concordant twins-would have a chance to be notified at the
diagnosis of a second cancer and in including them we should therefore have made
a selection. Nine pairs of concordant twins had to be excluded for this reason.

The remaining material may be studied in two ways:

A: A retrospective study could be made by means of all the pairs known
including those of which one partner had died before 1942, when our period of
observation actually began. In principle this may be permissible, but if we
compute the number of such pairs to be expected from the Danish birth rate for
twins and from the incidence figures for cancer and general mortality rates, we
find that only 10 per cent of the expected number have been notified to the registry.

B: A prospective study could be undertaken on the more limited material
of twin pairs of which both partners were alive when their first cancer occurred,
and after the opening of the registry. A computation based on the material of
the registry shows that out of this category about 20 per cent of the expected
number have been notified to the registry.

Because of this difference in quality between materials available for prospective
and retrospective study they cannot very well be combined into one, and for pur-
poses like the present the advantage of prospective studies are well known.

Thus, the material remaining for a prospective study comprises a total of
336 pairs distributed on the various categories as appears from the last column of
Table II (h). It should be noticed that the distribution by sex of 336 cases of
the various groups corresponds closely to that computed on the basis of Danish
figures for population and cancer incidence.

An analysis of the material for the prospective study gives figures as shown
in Table III. Here the material has been subdivided according to the period
elapsed between the occurrence of the two cancers. It appears that the computed
interval between the appearance of the two tumours shows no definite difference
from the observed interval either for monozygotic or for dizygotic twins.

The total of 7 observed monozygotic pairs of twins with cancer against a
computed number of 3 8 is somewhat higher than the observed figure for dizygotic
twins amounting to 7 against a computed number of 8 7. However, this difference
is not statistically significant.

As far as the site of tumour is concerned the present small material does not
justify conclusions to be drawn on any tendency for tumours to occur in the
same site in concordant twins. It is true that the material is small, but it should
be noticed that the tendency for tumours to occur at concordant site is in favour
of the dizygotic and not of the monozygotic twins being a total of 1 observed
case against 0 44 computed for monozygotic twins and 2 against 0 52 for dizygotic.

A further analysis of the pairs in which cancer occurred in monozygotic twins
of the prospective material does not give much impression of concordance with
regard to site of the tumour (Table IV).

329

A. NIELSEN AND J. CLEMMESEN

*4 00  ut  w~C

CO a

aq  00~~

0)D

1~ ~ ~ ~ ~ ~ ~ ~ ~ ~ ~~~~~1l

.9

C)

0 ^

O
bN

.d

C)

-4

0

0o

D-
0
0

?

oI

o ~1

E ~  p1

0<  E--

0

E--

.~0C

r 6

1.

o

IX Ct t-  o  n )~  ~  )~

* . . . *~

r    --0- _   o Ooooo o

00

1o00       I       o      Coo  I

0 C to

0-0

H  Q

"(.  ?

'4E

H FI  4

l0

rD 4  6.

-00 CO CO
000-

0000-

I _

c0 00, I a
4:~ oo <D  r

01

CO
I1

,--I

oI ioo

IIoI  o

0

o0 1      0   0cooIo

10   0 0   0 J

CO001   o  o  o I   I  o

(oo1~    oooo Io

It O o Qa

O_ 00   01  >

'~ C)

:> C)~._

D0 00 0 X CO

I1-

a

4C

0

LO

(4

*f  o

0
s [

t-- u   CO e I
G", O' O I

.   .   N

moo 00 =I Ca

0 0 0 -   ->
. .  *- eq r

IC I~

OOO ,--o

r ,x es I b

r:: <O (: MO?

- O CO
u-"q CO ,

0-0

010-.

-  - -
CaC

D7 14 1

4Q,~ 4 cm

oo0-o

C - _ 1  -n.

0 ) -I  oo o

I..

o0   0 0  0

-  10 I  m

I . I

00  0   0

00000   0

CO

I

0

H

0

0

* D  .   .   .   .   .

CD

O

O

0 4 .C.'   0 C4

C3 .    , 4  n

E?<   ce 4-  bo CD

0

8 ' ' '

v

330

r f t- = I -- CD I

+,- C4a n

0                    -

Pe 0

C)e.. . r- --       --

r

li0

01

I--

-0,--1

0~

co

> ,D

~0

. 0 0

A
,.-I '

- ^=n

0 d

-0 ^

._

9c

.$.b
Zs

o~
o4.Q

4e
q*C

I

*H;

IC

r-

r

r

O C
"-"a  c-

aq -I -4 1 Idq       C> C) C -4 0  1 -.4

-  Go       00 (D

0        .a M

t-4 ?     , ,

N         0

H--I

TWIN STUDIES IN DANISH CANCER REGISTRY

o o      o    o ooo     oo       oo o 0   o
? ;4

o00

0o0++

4.') o-  o     +o  + +c)+o  +  +   + + o   +

o~ .~

AD;4

i> oc

0

0  4

'og

>

ri  -  *

*   C)

?4e

EzVv

H

O4

C)o

o o C

R 2    2 o

V 0      0

C) o C

N    N -0Nt                  1 C4 t-  -4  - .4-
(D  oo  0  co  00 -* -4  Itt co4' 4' co

C)O  '  ?  0  ....  ~  '  ?  -..

IQ;   b-              0 '~    '

b  6

0~~~~

0> C  +  ~0  O C000  00  0  0   +o

+  0  ++  0  ++?  +  +  +

140. ~ ~ ~ ~

0- ~ ~ ~ ~ ~ ~ ~ ~ ~ ~ ~ ~ ~ ~

Cl ~  ~    o + +o +      +
;>

0                4 -s

C )    4 0~~~'   0D

bc~~~~~~~~c

:D 0           Ca

0 4   ~       0

o

0  0 ~ ~ 0  0    -.

C)                     I )

0  0          0  (D

13D w   0 ~ 0 ~ ~

C0o    00   0        0

U u   &4~~o"

?

CZD     t-    I- t-,.
Co       r N

0        COC   ON

C - CD      No

331

~-4

.5

-4-

B

r  m-4

Q)  -

o)

,?. 0

r.4 . 4

%4      Cd

(z)     0  .

0 p

IZ,      ce 't
I.-I    ?-z    ce

-       4-4-

-V   .2  0

HH

- 4

EH     4

EH

It
>, 0
-m C

A. NIELSEN AND J. CLEMMESEN

The finding of a pair showing two mammary cancers of the right breast is well
in keeping with the observations made by Busk from Cancerregisteret (1948)
that a relative of a patient with a cancer of the right breast will stand a heavier
risk of breast cancer in this side than in the left. It may also be mentioned that
gastric cancer and rectum cancer may both develop on the basis of familial
gastropolyposis of the intestine, so that one of the other pairs may not, after all,
be discordant as to the site of the tumour.

Thus, we have found no concordance of site which would not be expected
from other experience.

A corresponding analysis of the dizygotic twins shows one pair with cancer
of colon and rectum which may represent complete concordance. As far as uterine
carcinoma of the cervix is concerned we know from studies by Br0beck (1949)
and R0jel (1953) that this apparent concordance is likely to be due to exposure to
exogenic factors or sex habits identical for the two twins.

The retrospective material, which in itself would hardly justify conclusions,
show results corresponding to those from the prospective material (Table V).
Identity of site of the two tumours is found for tumours of breast and of the large
intestine and stomach as well as for the uterine cervix among the dizygotic.

It may, however, be of interest that cancer of the lip which usually is considered
exogenic occurs in both twins of a monozygotic male pair. Both were unskilled
labourers. The pair with two cancers in each partner may also be noticed.

With regard to concordance of site we have to admit that our material is too
limited in size to allow conclusions to be drawn.

SUMMARY

In our limited material we have found features suggestive of a feeble hereditary
tendency in neoplastic diseases. This tendency, however, is not statistically
significant and seems fully explained by the presence of such neoplastic diseases
as are known from other studies to be genetically influenced.

APPENDIX

Computations and their Background

For the computation of the expected number of cancer cases in a given popu-
lation with due regard to age we must know the number of years at risk for this
population distributed, according to age. If the number of years at risk for
the age X is denoted Lx, and the corresponding incidence of cancer Vx, we
find the expected number of cases by the formula:

00          00

LX XVx Vz     L5s+2.5 X V5s+2.5

X=0          8=0

supposing that we work on a basis of quinquennial age groups.

In our prospective study the period at risk for an individual pair of twins will
begin when the first partner is seen in hospital with cancer and duly notified as
twin, while the other twin has not suffered from cancer. The period at risk for the
pair will end either at July 1, 1955, or at the date, if earlier, when the second
twin is first seen in hospital or-if not so-dies with cancer.

332

TWIN STUDIES IN DANISH CANCER REGISTRY

?E0 O0 o      0 o  oo o o  o  +   +oooo

00

0   ?+o o  oo + o +      o  oo  o  o+-ooo

0

caa

mP~~~~~~~~~~ 03

I         - .  .-   -            -

U,

-              4~~~~~14'D
0o

,.cl

s   ,     z~~~~~~~~~~~~~C
4'

Mt~  ~~     ~ 4a Ca O j

.4'~~~~~~~~~~144

N   4  '       04  '

04

o   .   E

o   0   Ho  O  0  O .OQO

M2  0  0>           '0---P4(=4'1
~0~~~~~~~

~~~~~                           H

X ~ ~ ~ b      0          0   - ?=  - Ct =   :   :

Cs 0 GCa |     ]          g         B 0

N0sos g, *i^ 3^. 4 D -4

Ca  bo~~~~~

4Q                                Ca~~~~~0.~~

'"  .,> o  O   o o 0      o o 0  0  + o + 0 o

.'

0

404  ++    0+ + 0 0   +  ++   0   ++++ O

q )

., -4

p4

{il~ ~  ~    4 'm  .U,Sk'  l
0

?44 0

04  ~ ~ ~ ~       0      0

..        ~. o~ ? A  R  R  o   p

8 4~       14@  4 t  8     4     44 @

0 0        0 0  0  0  0          0     0VV

0 0   0 0   0   0   0  0  0 0   0~~~~~~~~~~~~~~~~~~~~~~~~~~~~~~~~~~~~~~~~~~C:
00         0  0 0  0 0     0      0

' 1     0 o   l   cz
~q          t e   I -

'      o CO 00  4o    CO    0  0

q      cc rI --  CD  .    co r'- - 00

333

0

o t

" 3

1HH

;.  PO  B

pq

E-,

H

4

A. NIELSEN AND J. CLEMMESEN

The incidence Vx will be given as the morbidity of cancer observed by the
Danish Cancer Registry for the years 1943-52 inclusive. Incidence is given for
cancer of all sites or for a group of sites according to the purpose.

In our retrospective study the period at risk for an individual pair of twins will
begin at their fifth anniversary, if both are alive. This arbitrary limit has been
set because of the uncertainty on mono- and dizygocity and other information in
cases when one partner died younger than five years old. The period at risk will
end at January 1, 1942, or at the date, if earlier, when one partner was first known
to suffer from cancer, possibly at death. The denotation Vx will stand for
the mortality of cancer in Denmark for the period 1931-40 as observed by the
National Health Service, since it will mostly be impossible to get information
retrospectively about a case of cancer which has been cured.

It is in the nature of a prospective study that the collection of information on
twin pairs will be unbiased, because it is impossible to foretell whether or not
a person will develop cancer at a later date. Contrarily, in the retrospective study
the very fact that a twin died some years earlier will reduce reliability of informa-
tion on mono- versus dizygocity as well as on the cause of death.

For the computations mentioned earlier in this paper we have estimated the
expected number of twin pairs of which one partner has died or developed cancer
before January 1, 1942, and of which both partners were alive on this date.

The following denotations were used:

A' is the probability that a twin, a, alive at the age of 5, will live to January 1,
1942, aged x year at this date and without cancer.

B] is the probability that a twin, a, alive on January 1, 1942, aged x year
at this date and without cancer will develop cancer before July 1, 1955.

Cab is the probability that a twin, a, both twins of the pair, a and b, alive
on January 1, 1942, and aged x year at this date without cancer, will develop
cancer before July 1, 1955, and that the other twin, b, will not develop cancer
before a.

Aa is taken as the proportion between the number of people living at the
census 1940 at various ages and the number of this generation living five years old,
which figure is found from a former census.

B" and Cab are computed from the morbidity of cancer observed by the
Danish Cancer Registry for the years 1943-52 inclusive, and from the mortality
of the Danish population in the period 1946-50.

We can compute the probability that both twins of a pair, a and b, are alive
on January 1, 1942, at an age of x > 5 years, and that a develops cancer before b
and before July 1, 1955, by the formula:

Aa. A  r . Cb

The corresponding probability for b to develop cancer before a will be computed
by the formula:

A a A>b (cba
Ax ? x .x

These two probabilites are equal if a and b are of identical sex.

In a similar way we can compute the probability that twin a lives to January 1,
1942, at an age of x and develops cancer before July 1, 1955, and that twin b has

TWIN STUDIES IN DANISH CANCER REGISTRY

died or had cancer before January 1, 1942, but more than 5 years old. This is found
by the formula:

AaBa (1     A).

In this case, too, we may reverse the sequence of a and b with the same effect
as given above.

From official Danish statistics we know the sex distribution of partners of living
new-born twin pairs. For 1906-40 32. 1 per cent are both males, 37.3 per cent
show one male and one female and 30 6 per cent two females. On the basis of the
mortality for 1895 to 1900 we can now compute the sex-distribution of living
twin pairs 5 years old, which is 31- 1 per cent two males, 37 3 per cent one male and
one female and 31 6 per cent two females.

Twin 1: male. Twin Il female      Twin 1: female.TwinII: male

FIG. 1.-Distribution by age of twin pairs alive without cancer on January 1, 1942.

Columns indicate fully notified cases on January 1, 1942. Curves indicate expected
number divided by five.

1

]

I

t

.L

I

335

I

336                A. NIELSEN AND J. CLEMMESEN

In the official statistics we find that 1.4 per cent of all births are twin births.
With due regard to the mortality we will find that at an age of 5 years the number
of twin pairs with both partners alive will have diminished to 1.15 per cent of
all births represented at that age by at least one living child.

Twin I: male. Twin II: female     Twin I: female. Twin II: male

10         I      I   I    I   I                I  I   I   I  I   I

6

4-

0      10 20 30 40 50 60 70 80 Age0      10 20 30 40 50 60 70 80
=     Twin I: male  Twin I1: male         Twin I:female. Twin lI:female
.4i14' !   1   1  1                                      I

2   I  I   I  i                     I I   :   I,   IX          1
' 12-

iox-
<10

6-
4-
2-

0   10 20 30 40 50 60 70 80Age 0       10 20 30 40 50 60 70 80

FIG. 2.-Distribution by age of twin pairs of which one partner (II) has died or developed

cancer before January 1, 1942. Columns indicate cases fully notified on January 1, 1942.
Curves indicate expected number divided by five.

For census figures we know the number aged 5 years at the time of census.
From these numbers and from the values already given it is possible to compute
the expected number of twin pairs distributed according to age on January 1,
1942, of which one partner will get cancer in the period from January 1, 1942,
to July 1, 1955 (a) if both were alive January 1, 1942, and (b) if one has had cancer
before this date. The expected curves are given in Fig. 1 and 2.

REFERENCES

BR0BECK, O.-(1949) 'Heredity in Cancer Uteri.' Arhus. (JUniversitetsforlaget).

BUSK, T.-(1948) Ann. Eugen. Lond., 14, 213.-(1952) Proceedings of the Second

National Cancer Conference, 2, 1087. Cincinnati. Amer. Cancer Soc.
Idem, CLEMMESEN, J. AND NIELSEN, A.-(1948) Brit. J. Cancer, 2, 156.

JACOBSEN, O.-(1946) 'Heredity in Breast Cancer.' K0benhavn. (Nyt Nordisk Forlag.)
MOSBECH, J.-(1953). "Heredity in pernicious anaemia" K0benhavn (Munksgaard)

R0JEL, J.-(1953) 'The Interrelation between Uterine Cancer and Syphilis'. Kobenhavn

(Nyt Nordisk Forlag.).

VIDEBAEK, AA.-(1947) 'Heredity in Human Leukemia and its Relation to Cancer'.

K0benhavn. (Munksgaard).

Idem AND MOSBECH, J.-(1954) Acta med. scand., 149, fasc. II, 137.

				


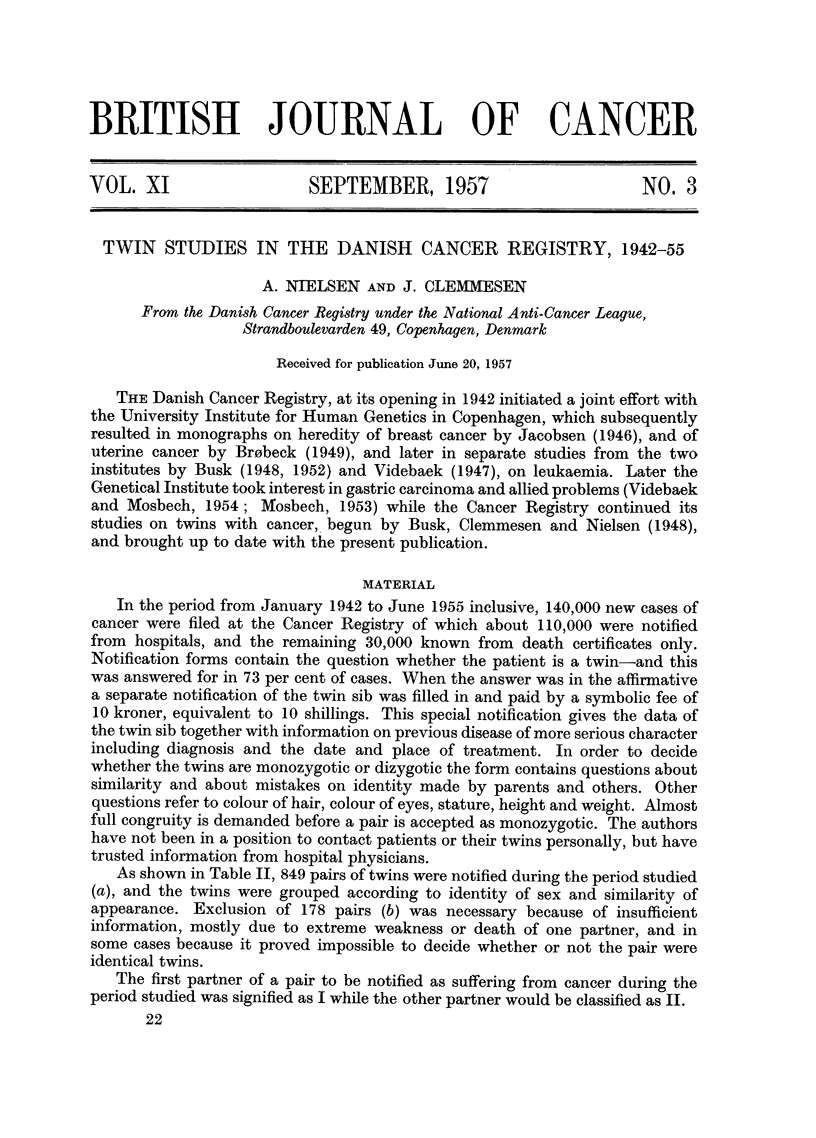

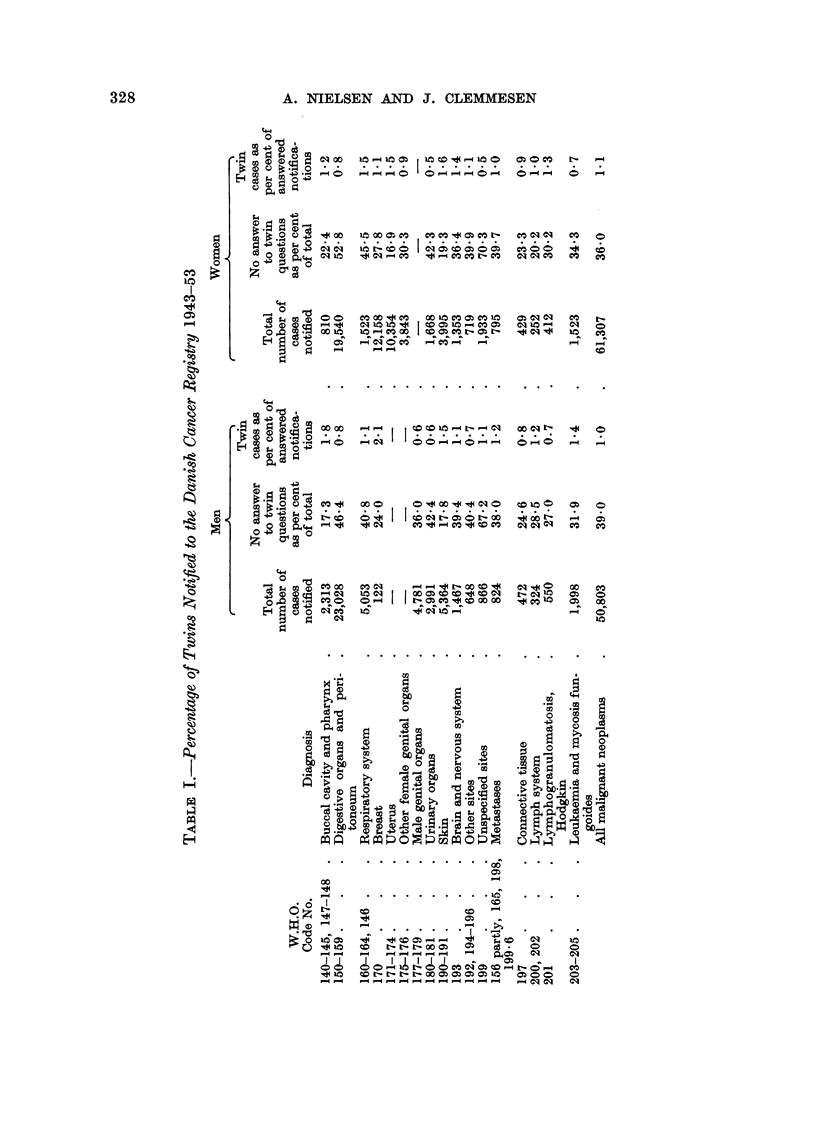

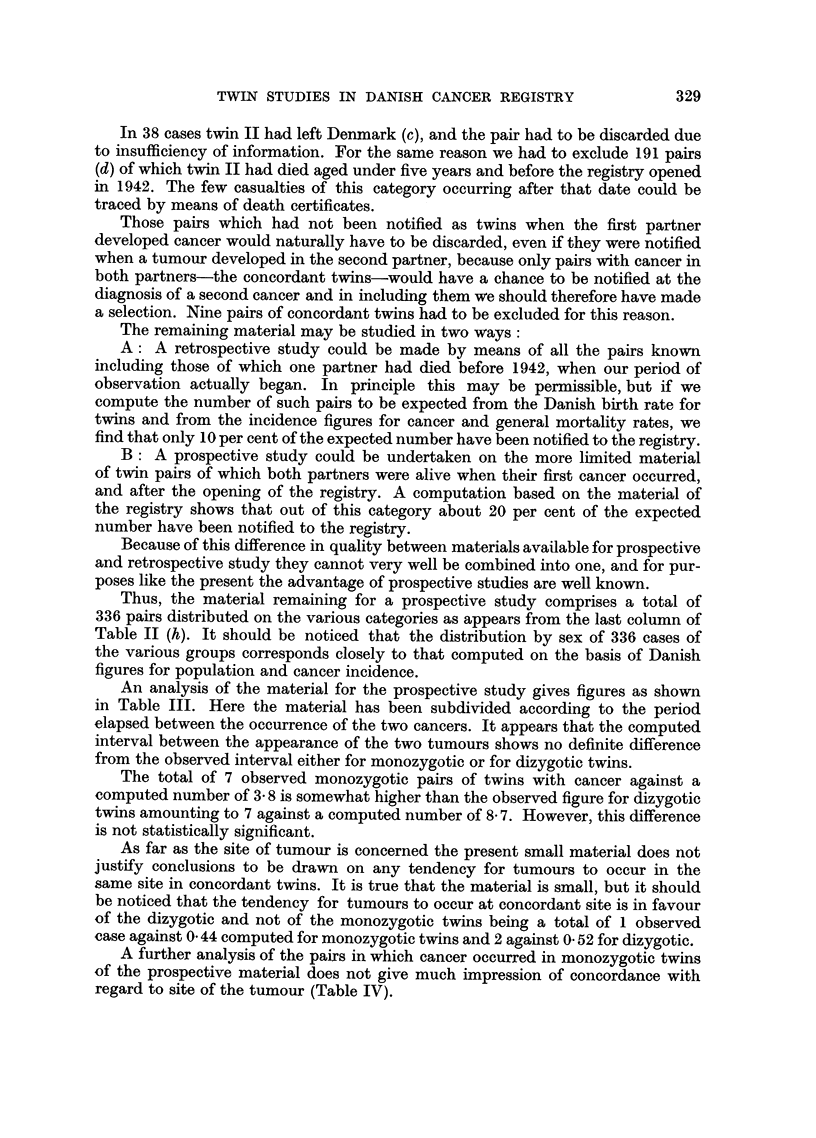

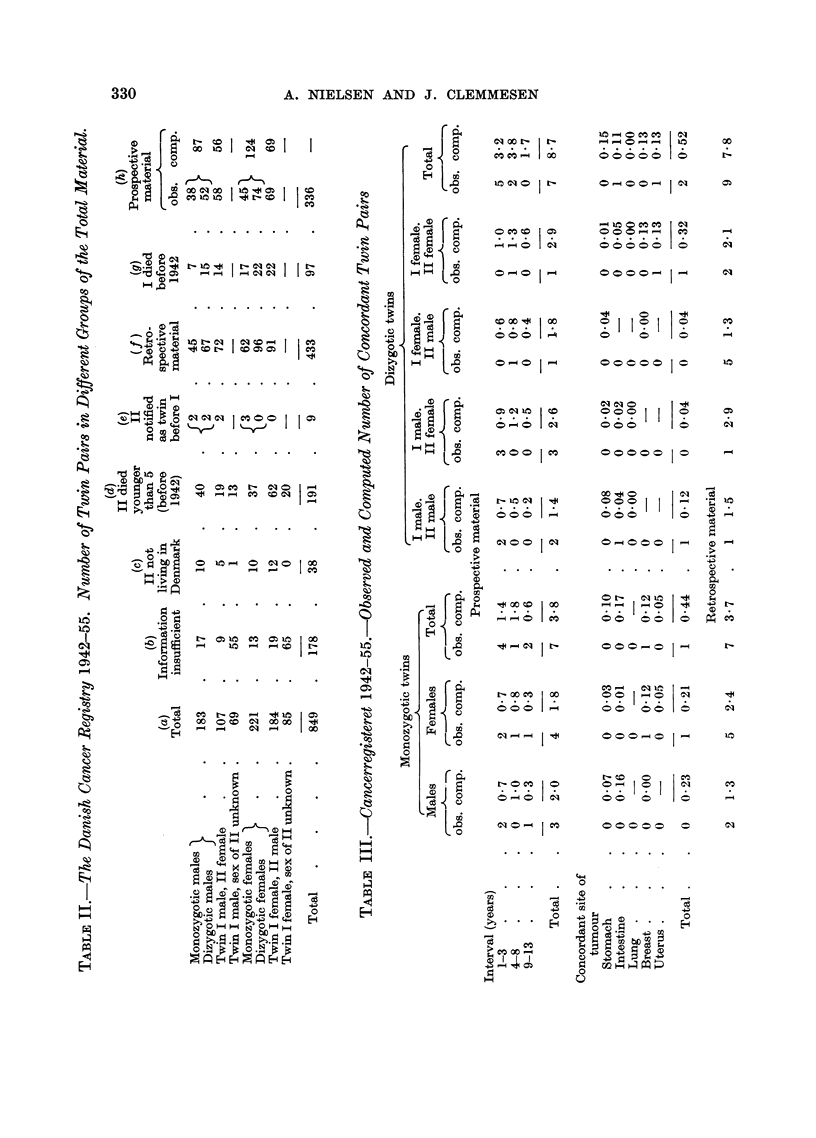

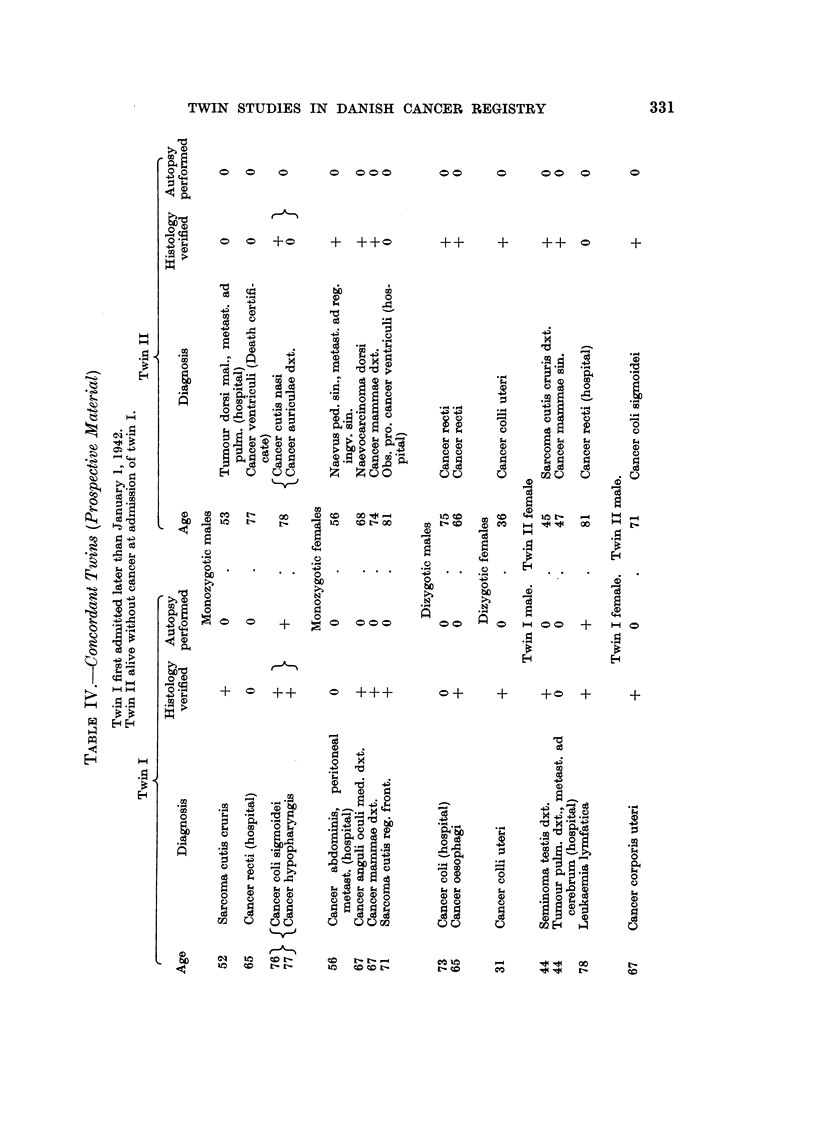

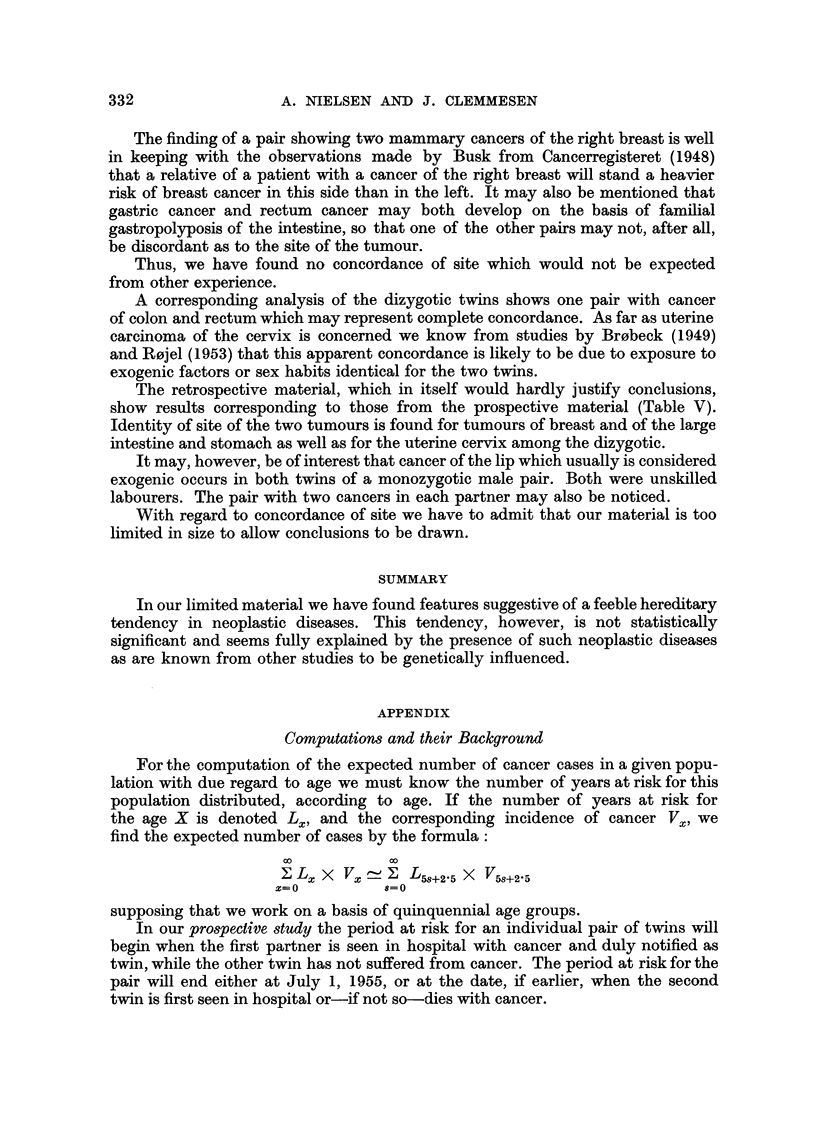

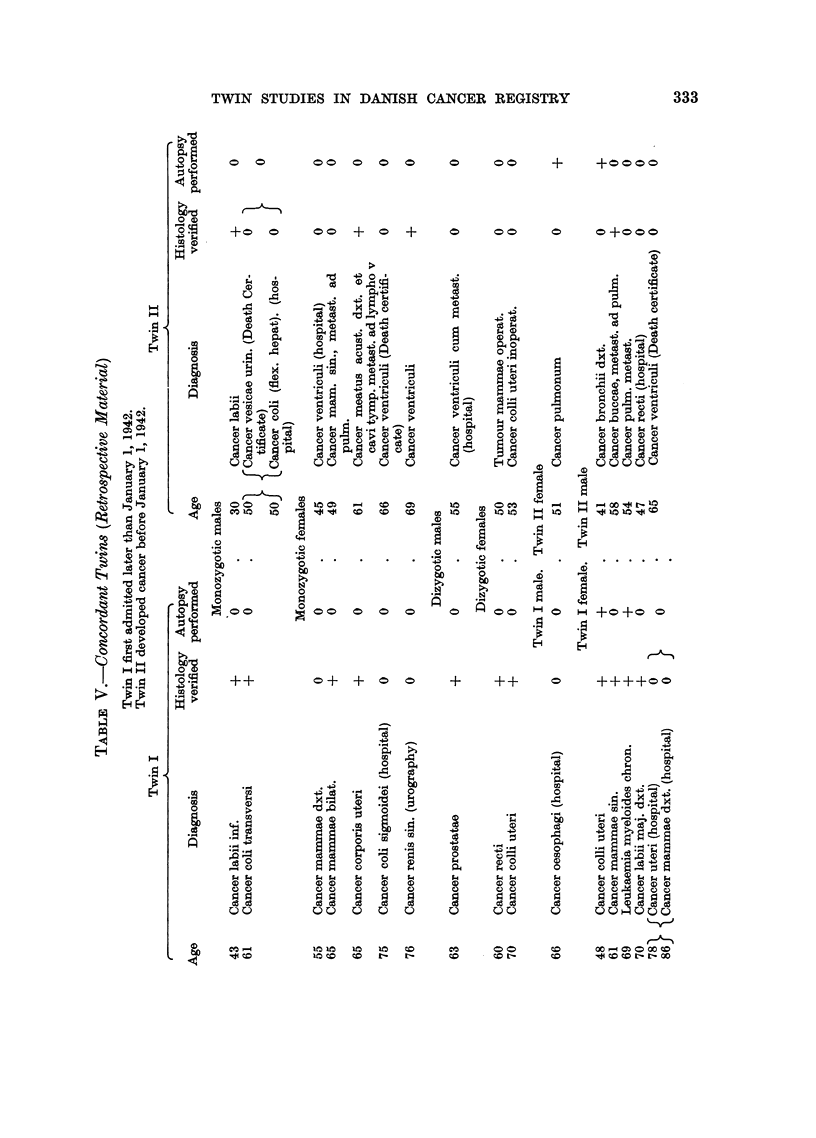

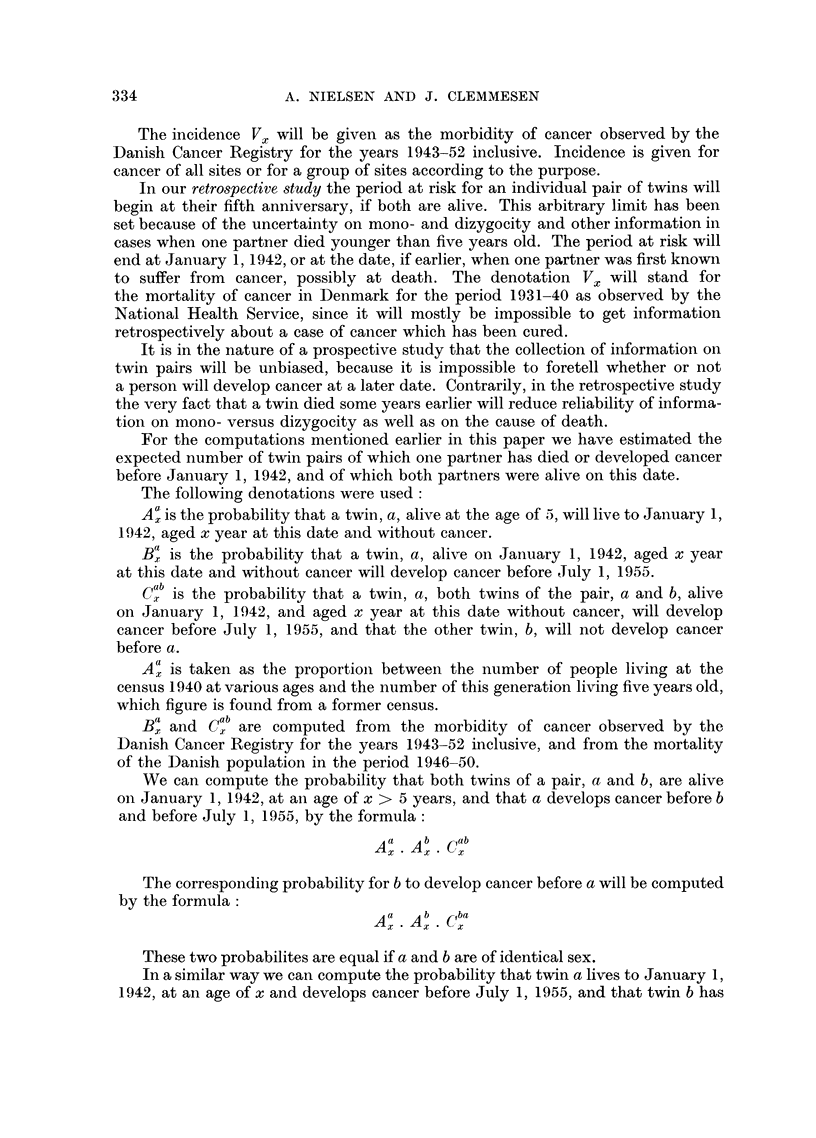

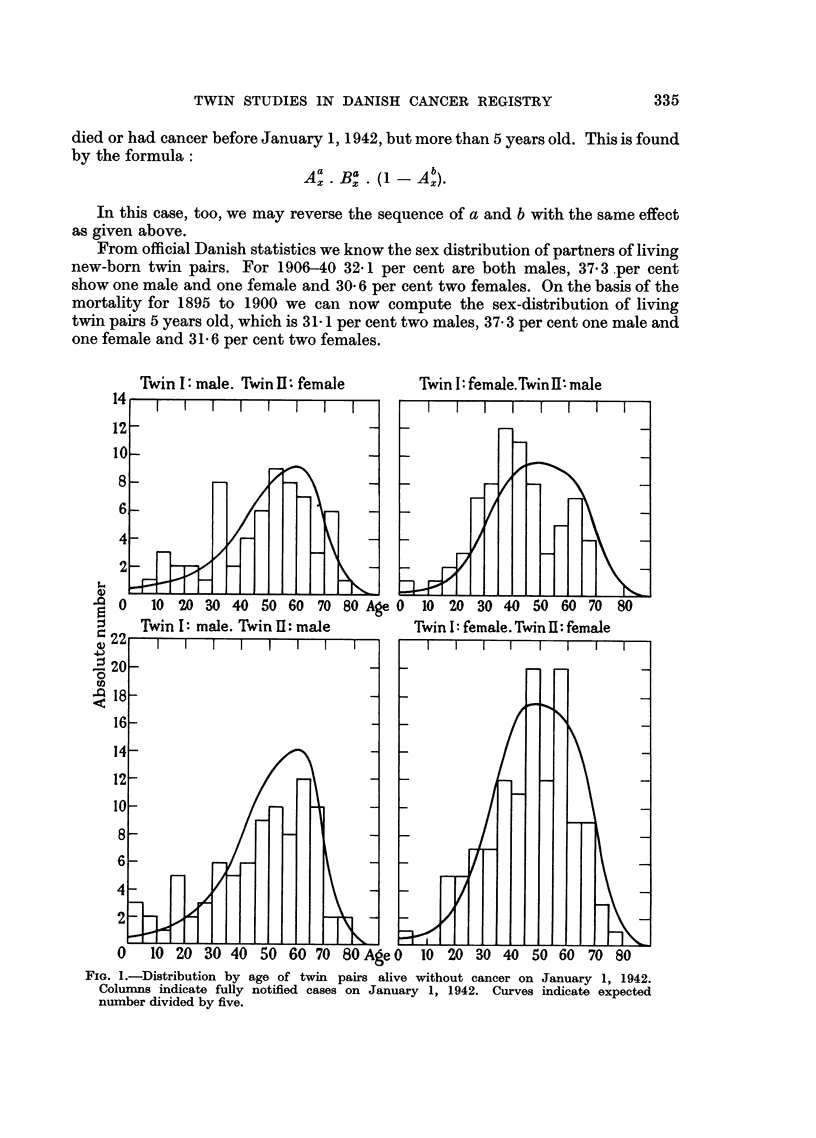

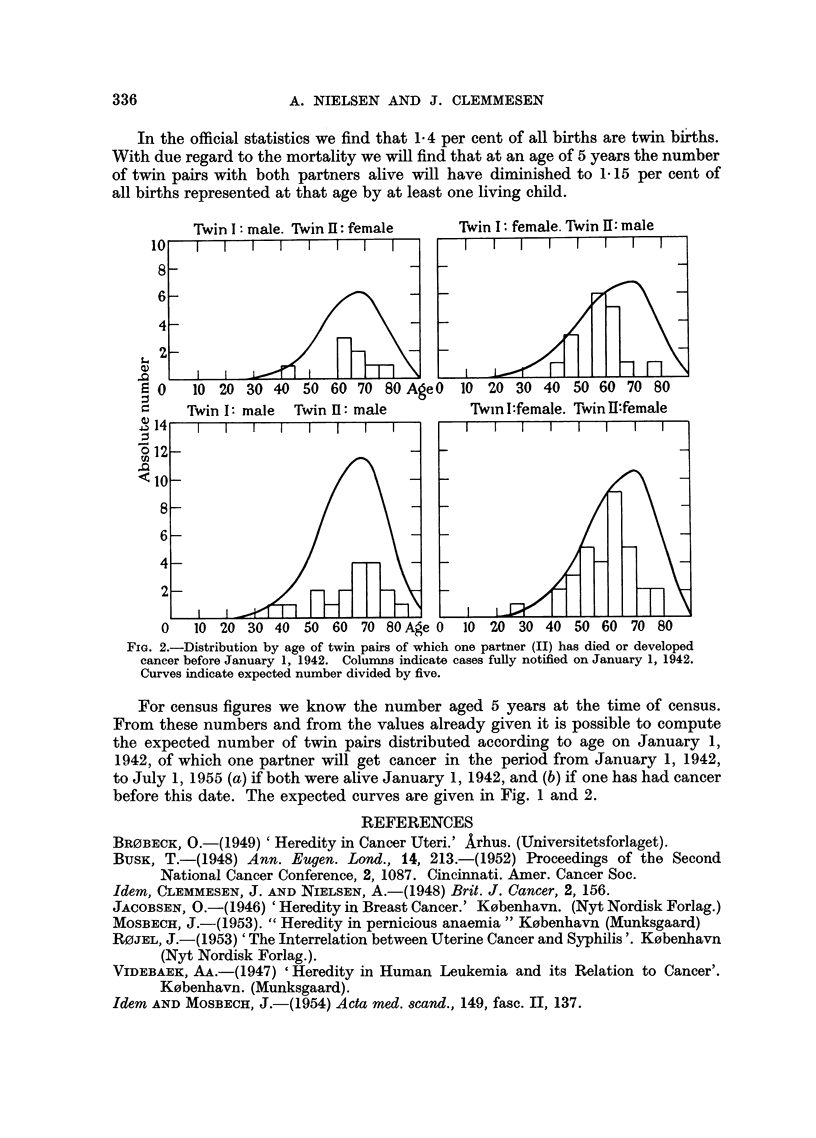

